# Gualou Guizhi Decoction Improves Glucose Metabolism and Alleviates Microglia-Associated Inflammation after Cerebral Ischemia

**DOI:** 10.1155/2022/9438250

**Published:** 2022-10-22

**Authors:** Jizhou Zhang, Jing Han, Jun Zou, Chang Jiang, Chunquan Zhou

**Affiliations:** ^1^Institute of Materia Medica, Fujian Academy of Chinese Medical Sciences, Fuzhou, Fujian 350003, China; ^2^Pharmacy College, Fujian University of Traditional Chinese Medicine, Fuzhou, Fujian 350122, China

## Abstract

**Background:**

The classical prescription Gualou Guizhi decoction (GL), a mixture of *Radix Trichosanthis*, *Ramulus Cinnamomi*, *Radix Paeoniae Alba*, *Radix Glycyrrhizae*, *Zingiberis Rhizoma Recens,* and *Fructus Ziziphus Jujuba*, was clinically used in the treatment of limb spasms after stroke and has achieved remarkable therapeutic effects. However, the underlying mechanism still needs to be further explored.

**Methods:**

Cerebral ischemia/reperfusion (CI/R) in Sprague-Dawley rats was induced by middle cerebral artery occlusion followed by filament removal. GL was intragastrically administered once daily for 7 or 14 consecutive days. The effect of GL on neurobehavioral impairment was evaluated. ^18^F-FDG micro-PET imaging was used to detect the effects of GL on glucose utilization in neural cells after CI/R. Immunohistochemical staining of glucose transporter 1 (Glut-1), glial fibrillary acidic protein (GFAP), and ionized calcium-binding adaptor molecule-1 (Iba-1) was further performed to show the effects of GL on cerebral glucose transport and the activation of inflammatory-related glial cells. Markers related to the microglial subtype were also assessed to investigate the effects of GL on microglia polarization.

**Results:**

Neurological deficits induced by CI/R were significantly improved by GL administration. GL restored the glucose uptake in the ischemic hemisphere. Glut-1, the major glucose transporter in the brain, was significantly increased after GL treatment. Moreover, GL mitigated the activation of astrocytes and microglia after CI/R. Furthermore, GL significantly decreased proinflammatory M1-type microglial markers TNF-*α* and iNOS, while increasing anti-inflammatory M2 microglial markers CD206 and Arg-1.

**Conclusion:**

GL enhanced the uptake and utilization of glucose in neural cells after CI/R. It exerted significant anti-inflammatory effects by regulating the polarization of microglia. These results provided further evidence supporting the clinical application of GL in the treatment of cerebral ischemic stroke.

## 1. Introduction

Stroke is the second leading cause of death globally with an annual mortality of around 5.5 million. It is also the leading cause of disability worldwide. Chronic disability occurs in 50% of stroke survivors [1]. Ischemic stroke accounts for nearly 80% of all strokes. It is due to cerebrovascular obstruction that reduces cerebral blood flow, resulting in focal cerebral ischemia and hypoxia. Its high morbidity rate, high disability rate, and high recurrence rate have caused a heavy economic and social burden on society and families. Acute treatment of stroke has improved considerably in the past two decades with the introduction of stroke units and the use of thrombolysis and/or thrombectomy. However, nearly 75% of ischemic stroke patients have varying degrees of neurological dysfunction [2], of which poststroke spasms are very common. Spasticity occurs in approximately 43% and 38% of patients 6 and 12 months after stroke, respectively [3]. The spasms significantly reduce the motor function of the affected upper extremity.

Gualou Guizhi decoction (GL) is an adjunctive alternative therapy in traditional Chinese medicine to relieve the symptoms of limb spasms. The decoction is documented in *Jingui* Yaolüe written by Han Dynasty doctor Zhang Zhongjing more than 1800 years ago. Clinically, GL has been applied to the treatment of poststroke limb spasms and has achieved a remarkable curative effect, which can significantly improve limb mobility. In recent years, research has been conducted to uncover the underlying mechanism of GL in the treatment of poststroke spasticity. GL showed a significant neuroprotective effect in an NMDA-induced hippocampal primary neuron injury model *in vitro*, as well as in a rat transient middle cerebral artery occlusion (MCAO) reperfusion model *in vivo* [4]. Its mechanism of action may involve regulating the balance of excitatory amino acids in brain tissue [5–7], anti-inflammation [8], antiapoptosis [9], and antioxidant activity [10].

Glucose is one of the three major energy substances in the human body. After cerebral ischemia, neurons' glucose utilization is one of the key factors for neural survival and repair [11]. The utilization of glucose in the brain directly reflects the viability of neural cells after cerebral ischemia. Therefore, it is closely related to the recovery of limb spasms after stroke. However, the effect of GL on glucose metabolism in the brain after cerebral ischemia has not been reported.

Positron emission tomography (PET) is a type of functional imaging used to image the metabolic or physiological functions of the body. It is a minimally invasive imaging procedure that not only visualizes metabolic changes in tissue but also determines its localization [12]. The positron-emitting radiopharmaceuticals ^18^F-fluorodeoxyglucose (^18^F-FDG) can be used for *in vivo* imaging of glucose metabolism in the brain. Using this technique, the efficacy of drugs on changes in brain energy metabolism after cerebral ischemia can be evaluated noninvasively.

Microglia are innate immune cells in the central nervous system (CNS) that are rapidly activated after cerebral ischemia. Activated microglia have a dual role in ischemic injury [13]. Previous studies have explored the neuroprotective effect of GL in an *in vitro* cultured BV2 microglia neuroinflammation model [9, 14]. However, the effect of GL on cerebral ischemia-induced microglia activation *in vivo* has not been demonstrated.

In this study, ^18^F-FDG micro-PET imaging was used to observe the effect of GL on brain glucose metabolism in a rat model of middle cerebral artery occlusion (MCAO)/reperfusion. The regulation of GL on microglia activation and polarization was further analyzed. Collectively, the current study complements the evidence for the neuroprotective effect of GL on cerebral ischemia-reperfusion and uncovers a possible mechanism of GL to treat limb spasms after cerebral ischemic stroke.

## 2. Materials and Methods

### 2.1. Animals

Male Sprague-Dawley (SD) rats were purchased from SLAC Laboratory Animal Co. Ltd., Shanghai, China (SCXK (Hu) 2019–0002). Rats were housed under a 12-hour light/dark cycle (light time: 8 : 00–20 : 00) with free access to a standard diet and water. Focal cerebral ischemia/reperfusion was induced after adaptive feeding to a body weight of 260–300 g. The study complied with the international animal experimentation laws and was approved by the Ethics Committee of Fujian Academy of Chinese Medical Science, China (approval no. FJATCM-IAEC2020011).

### 2.2. Transient Middle Cerebral Artery Occlusion (MCAO)/Reperfusion Model

The focal cerebral ischemia/reperfusion model was induced as described in our previous study [15]. Rats were anesthetized with 2–3% isoflurane. After anesthesia, a vertical midline incision was made in the neck in order to isolate the right common carotid artery (CCA), external carotid artery (ECA), and internal carotid artery (ICA). The ECA and its branches were then cauterized and coagulated, and the distal end of the ECA was ligated. The CCA and ICA were temporarily clamped, and a silicone-coated nylon monofilament (Guangzhou Jialing Biotechnology Co., Ltd., Guangzhou, China; L3600 or L3800) was inserted into the ICA through the stump of ECA until slight resistance occurred. At this point, the tip of the monofilament was approximately 18 mm from the bifurcation of the CCA and ECA, just occluding the middle cerebral artery (MCA). After 120 min, animals were re-anesthetized with isoflurane, and the filament was removed from the ICA to allow MCA reperfusion. Rats in the sham group underwent all surgical procedures except for CCA occlusion.

The neurological behavior of the MCAO model was assessed 24 hours after reperfusion to screen animals successfully modeled using the 5-point system developed by Longa et al. [16] as follows: 0, no deficit; 1, difficulty in fully extending the contralateral forelimb; 2, difficulty in routing; 3, circling to the contralateral side; and 4, falling to the contralateral side. Rats scoring 1–3 were included in the following tests.

### 2.3. Experimental Grouping and Drug Administration

Twenty-four hours after reperfusion, all the selected animals (except those in the sham group) were divided into the vehicle group and the GL group according to their neurological behavior scores to ensure the consistency of the degree of cerebral ischemic injury between groups. Rats in the GL group were gavaged with GL, while rats in the vehicle and sham groups were gavaged with saline once a day for 7 consecutive days. For rats undergoing micro-PET detection, treatments were performed for 14 consecutive days.

### 2.4. Preparation of Gualou Guizhi Decoction

The GL is composed of *Trichosanthes kirilowii Maxim.* (*Cucurbitaceae: Radix Trichosanthis*), *Cinnamomum cassia Presl.* (*Lauraceae: Ramulus Cinnamomi*), *Paeonia suffruticosa Andr*. (*Ranunculaceae: Radix Paeoniae Alba*), *Glycyrrhiza uralensis Fisch.* (*Leguminosae: Radix Glycyrrhizae*), *Zingiber officinale Roscoe.* (*Zingiberaceae: Zingiberis Rhizoma Recens*), and *Ziziphus jujuba Mill.* (*Rhamnaceae: Fructus Ziziphus Jujuba*). The proportion of each medicine is as follows: 30 g:9 g:15 g:6 g:9 g:30 g. The standard granules of the ingredients were purchased from Beijing Tcmages Pharmaceutical Co., LTD (Beijing, China). The granules of each ingredient were mixed in a 100°C boiling water (120 mL/dose) bath for 30 minutes to prepare a decoction. The oral administration dose is 10 mL/kg, which is equivalent to 6 g/kg of herbal medicine. The dosage is 5 times the clinical use for humans, which is calculated based on the body surface area of humans and rats.

### 2.5. Micro-PET *In Vivo* Imaging

Micro-PET *in vivo* assays were performed at 3, 7, and 14 days after reperfusion. Rats were placed in an anesthesia box, preanesthetized with 3% isoflurane at an oxygen flow rate of 3 L/min, and then injected with a 0.2 mL (37 Mbq) ^18^F-FDG labeled probe via the tail vein. Forty minutes after injection, rats were loaded into Siemens Inveon Micro-PET/CT (Munich, Germany) for scanning and three-dimensional image reconstruction. The micro-PET/CT has a resolution of 1.5 mm, a cylinder diameter of 5.7 cm, and a transaxial field of view (FOV) of 8.5 cm. The rats were fixed in the prone position in the FOV center and continuously anesthetized with 1.5% isoflurane at 2 L/min. Rats were scanned with CT (FOV: 8.5 × 5.7 cm) for 5 min, and then data were collected for 10 min. CT data were used for attenuation correction and anatomical delineation of images.

Image analysis was performed using Inveon Research Workplace (Siemens AG, Munich, Germany). Based on the PET/CT fusion image, we used the ROI tool to draw a region of interest (ROI) around the CT image area with a known mu value, quantify the uptake value of the probe in the brain, and calculate the average standard uptake value (SUV). After ischemia, the uptake value of the probe decreased. We calibrate the volume for low uptake values by adjusting the threshold of the color histogram bat to match the low mu value field.

### 2.6. Neurobehavioral Assessment

Rats were neurologically assessed using the modified neurological severity score (mNSS) and beam balance test at 7 and 14 days after MCAO surgery.

The mNSS was based on [17], which includes an assessment of an animal's motor, sensory, balance, and reflex functions. Assessments are graded on a scale of 0 to 14.0: normal neurological function, 1–5: slight neurological impairment, 6–10: moderate neurological impairment, and 11–14: severe neurological impairment.

The beam balance test assesses the coordination and balance of an animal's limbs. Rats were placed on a 1.5 cm wide by 30 cm long balance beam 30 cm above the platform [18]. Behavioral performance of animals on the beam is scored on a scale of 0–6: 0: balance, with postural stability; 1: grab the edge of the beam; 2: hold the beam, one limb falls off the beam; 3: hold the beam, both limbs fall off the beam, or rotate around the beam (>60 s); 4: tried to balance on the beam but fell (>40 s); 5: tried to balance on the beam but fell (>20 s); and 6: fall or try to balance or hang on the beam (<20 s). A higher score means more severe impairment.

### 2.7. Immunohistochemical Staining and Image Analysis

Seven days after reperfusion, five rats in each group were subjected to immunostaining. Rats were anesthetized with isoflurane and perfused with 0.9% saline, followed by adding 4% paraformaldehyde solution from the left ventricle until the limbs were rigid. Brains were dissected and immersed in paraformaldehyde-fixed solution containing 20% sucrose, followed by 0.1 M sodium phosphate buffer containing 30% sucrose until the brain sank to the bottom. After that, according to the stereotaxic map of the rat brain, 30 *μ*m-thick frozen coronal brain sections with a coronal plane of 1.7–1.0 mm (striatum) before the anterior fontanelle were selected and sliced using a freezing microtome. Sections were preserved in 0.05 M sodium phosphate buffer containing 30% sucrose and 30% ethylene glycol at −20°C prior to staining.

Immunohistochemical staining was performed using the ready-to-use rapid immunohistochemical MaxVision™ detection kit (Maixin Biotech. Co., Ltd., Fuzhou, China, KIT-5005). Sections were washed 3 times with 0.01 M PBS and then incubated in 3% hydrogen peroxidase for 10 min to inhibit endogenous peroxidase activity. The sections were then incubated in Rb pAb to Glut-1 (1 : 100, Servicebio, GB11215), Rb pAb to GFAP (1 : 1000, ab7260), Ms mAb to Iba-1 (1 : 100, Abcam, EPR16589), Rb pAb to TNF-*α* (1 : 500, Abcam, ab6671), Rb pAb to CD206 (1 : 1000, Abcam, ab64693), and Rb pAb to Arg-1 (1 : 200, Abcam, ab91279) antibodies, respectively. After overnight incubation at 4°C, sections were further incubated with HRP-polymeranti-rabbit or anti-mouse IgG working solution for 15 min. After each incubation, sections were washed with 0.01 M PBS for 3 times. Afterwards, sections were stained with diaminobenzidine (DAB) for 5–10 min, then washed and mounted on slides, dehydrated, cleared, and mounted with neutral glue. The slides were viewed under an inverted microscope.

To quantify the immunoreactivity intensity of Glut-1, Iba-1, and GFAP, five nonoverlapping fields from each rat were captured for measurement. The intensity of target protein immunoreactivity was represented by the integrated density (IntDen) of the entire field and analyzed using Image-J 1.51 K software (National Institutes of Health, Bethesda, MD, USA). The immunoreactivity intensity of each rat was calculated from the mean IntDen of all acquired images using the same threshold.

### 2.8. Immunofluorescence Staining

Immunofluorescence staining was performed on floating sections. Sections were washed in PBS and then incubated in blocking buffer for one hour at room temperature. Afterwards, in order to label the different polarized phenotypes of Iba^+^ microglia, the sections were incubated in Ms mAb to Iba-1 (1 : 100, Abcam, EPR16589) and other antibodies including Rb pAb to TNF-*α* (1 : 500, Abcam, ab6671), Rb pAb to iNOS (1 : 200, Abcam, ab15323), Rb pAb to CD206 (1 : 1000, Abcam, ab64693), and Rb pAb to Arg-1 (1 : 200, Abcam, ab91279) antibodies at 4°C overnight, respectively. Sections were then incubated in fluorescent secondary antibodies for one hour at 37°C in the dark. After each incubation, sections were washed 3 times with 0.01 M PBS. Finally, the sections were mounted on slices and observed under a laser scanning inverted microscope (LEICA DMi8, Buffalo Grove, IL, USA).

### 2.9. Enzyme-Linked Immunosorbent Assay (ELISA)

The dissected sections of the ischemic hemisphere were homogenized (10% w/v) in PBS and centrifuged at 12,000 r/min for 15 min. The supernatants were used for the determination of Iba-1, TNF-*α*, iNOS, CD206, and Arg-1 protein levels using ELISA kits (Jiangsu Meibiao Biotechnology Co., Ltd). The ELISA was performed using a microplate reader (Bio Tek Synergy H1, Santa Clara, CA, USA) according to the manufacturer's manual.

### 2.10. Real-Time PCR Assay

Two hours after the last GL administration, the rats were sacrificed under deep anesthesia, and the ischemic cortex and striatum were removed for RT-PCR. Total RNA was extracted using Trizol, and RNA concentration and purity were determined with an ultramicro spectrophotometer (Nanodrop2000, Thermo, MA, USA). Afterwards, reverse transcription was performed using First Strand cDNA Synthesis Kit (Servicebio Technology Co., Ltd, Wuhan, China). Real-time PCR was carried out on an ABI StepOne plus real-time PCR device (Applied Biosystems, CA, USA), using the 2x Under SYBR Green qPCR Master Mix (High Rox) reaction system. The amplification procedure was as follows: predenaturation at 95°C for 10 min (95°C, 5 s ⟶ 60°C, 30 s) ×45 cycles. The melting curve was measured at 65°C ⟶ 95°C with a temperature increase of 0.3°C per 15 s. The relative target gene expression was quantified using the 2^−ΔΔCT^ method. All primers were synthesized by Servicebio (Wuhan, China). The primer information is given in Table 1.

### 2.11. Statistical Analysis

All analyses were performed using Graphpad Prism 8.0 software (Graphpad Software, San Diego, CA, USA). Data are presented as mean ± standard error of the mean. Comparisons between groups were assessed by one-way analysis of variance followed by Bonferroni's test. *P* values less than 0.05 were considered statistically significant.

## 3. Results

### 3.1. GL Increased the Glucose Uptake in the Rat Brain after MCAO Surgery

**Figure 1:**
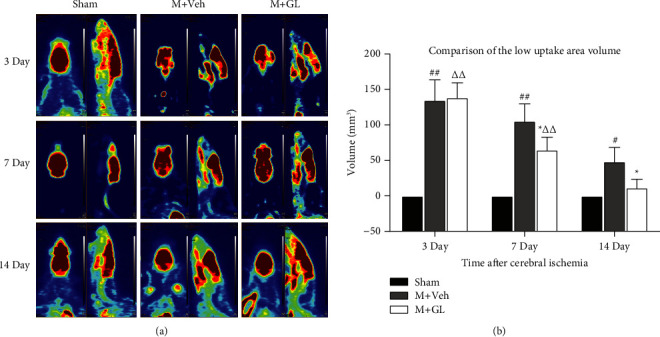
Effects of GL on ^18^F-FDG uptake in rats with cerebral ischemia-reperfusion injury. (a) Representative micro-PET images of each group were obtained on days 3, 7, and 14 after cerebral ischemia reperfusion. (b) Statistical analysis shows the ^18^F-FDG low uptake volume. Data were obtained from 3 rats in each group. Sham: sham-operated rats, M + Veh: MCAO rats treated with vehicle, and M + GL: MCAO rats intragastrically administered with GL. Results were presented as means ± SE. ^#^*P* < 0.05 and ^##^*P* < 0.01, M + Veh group compared with the sham group; ^*∗*^*P* < 0.05, M + GL group compared with the M + Veh group; ^ΔΔ^*P* < 0.01, M + GL group compared with the sham group.

GL was intragastrically administered after CI/R injury induced by MCAO surgery. To detect brain glucose uptake, three rats in each group were selected after neurobehavioral assessment for micro-PET imaging. The results showed that the brain structure of the sham group was intact, and no areas of reduced uptake of ^18^F-FDG were detected. In contrast, in rats with CI/R injury, the uptake of ^18^F-FDG was significantly reduced in the striatum and surrounding regions. The volume of the area with decreased ^18^F-FDG uptake was significantly reduced as the time after ischemia/reperfusion was prolonged to 7 and 14 days after reperfusion. At that time point, GL treatment significantly reduced areas of low ^18^F-FDG uptake compared with the vehicle group (Figure 1), indicating that GL can significantly improve glucose uptake in neural cells following CI/R injury.

### 3.2. GL Ameliorated the Neurobehavioral Impairment Caused by CI/R

**Figure 2:**
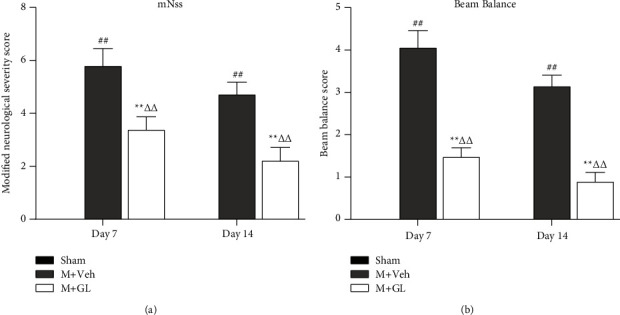
GL improved the neurological deficit of rats after cerebral ischemia/reperfusion. (a) Modified neurological severity score and (b) beam balance score. Data were obtained from 12 rats in each group. Sham: sham-operated rats, M + Veh: MCAO rats treated with vehicle, and M + GL: MCAO rats intragastrically administered with GL. Results were presented as means ± SE. ^##^*P* < 0.01, M + Veh group compared with the sham group; ^*∗∗*^*P* < 0.01, M + GL group compared with the M + Veh group; ^ΔΔ^*P* < 0.01, M + GL group compared with the sham group.

After CI/R, the neurobehavioral function of rats was impaired, and the mNSS score was significantly increased (*P* < 0.01). After seven days of GL administration, mNSS scores decreased significantly (*P* < 0.01), indicating an improvement in neurobehavioral deficits (Figure 2(a)). The beam balance test assesses the coordination and balance of the animal's limbs. The results (Figure 2(b)) showed that the beam balance scores of the GL group were significantly lower than that of the vehicle group. The improvement was more remarkable after 14 days of treatment (*P* < 0.01). These results suggest that GL can significantly improve limb function in animals after cerebral ischemia.

### 3.3. GL Increased Glut-1 Expression in the Cerebral Ischemic Area

**Figure 3:**
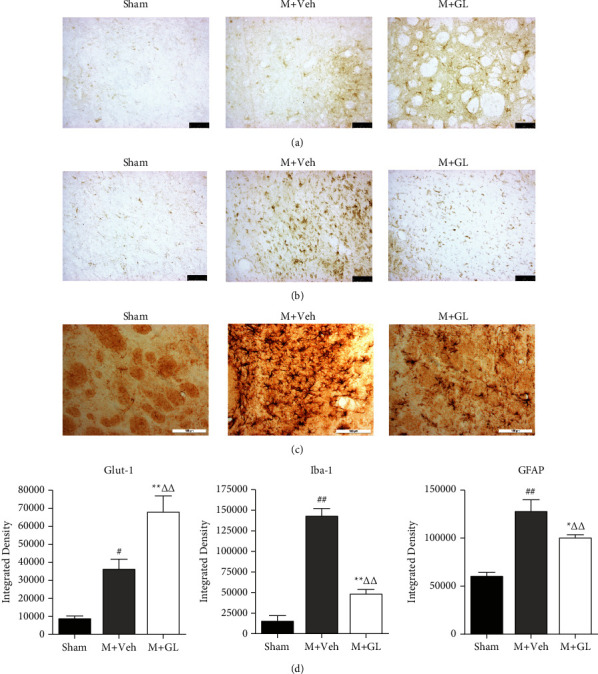
The effects of GL treatment on Glut-1 expression and the hallmarks of microglia and astrocytes after cerebral ischemia/reperfusion. Images were captured on the adjacent region of the ischemic area. Integrated density data were obtained from 5 animals in each group. (a) Glut-1, (b) Iba-1, (c) GFAP, and (d) quantification of integrated density of Glut-1, Iba-1, and GFAP. Sham: sham-operated rats, M + Veh: MCAO rats treated with vehicle, and M + GL: MCAO rats intragastrically administered with GL. Results were presented as means ± SE. ^#^*P* < 0.05 and ^##^*P* < 0.01, M + Veh group compared with the Sham group; ^*∗*^*P* < 0.05 and ^*∗∗*^*P* < 0.01, M + GL group compared with the M + Veh group; ^ΔΔ^*P* < 0.01, M + GL group compared with the sham group. Bar in (a) and (b): 75 *μ*m and bar in (c): 100 *μ*m.

Glucose transporter 1 (Glut-1) is the major glucose transporter in the central nervous system. Immunohistochemical staining showed that only a small amount of Glut-1 was expressed in the striatum of the sham group. However, after CI/R injury, the expression of Glut-1 in the ischemia-adjacent area was significantly increased. After GL treatment, the expression of Glut-1 was significantly elevated compared with the vehicle group (Figure 3(a)).

### 3.4. The Effects of GL Treatment on Iba-1 and GFAP Expression after CI/R

Iba-1 and GFAP are markers of activated microglia and astrocytes, respectively, in the nervous system. Immunohistochemistry staining showed that after CI/R injury, Iba-1 and GFAP-positive cells in the striatum around the ischemic area were activated, with enlarged cell bodies and elongated branches. Moreover, their immunopositivity increased significantly. GL treatment reverses the increase in immunoreactivity of Iba-1 and GFAP (Figures 3(b) and 3(c)).

### 3.5. Effect of GL on the Phenotype of Microglia after CI/R

**Figure 4:**
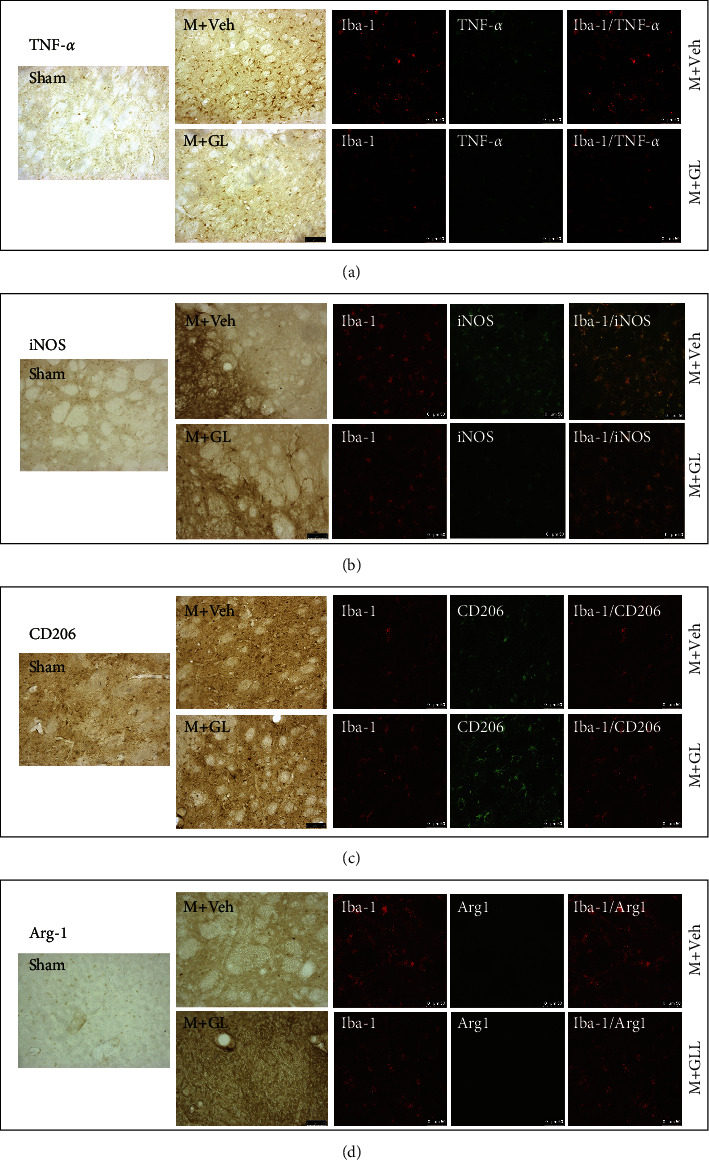
The representative images of immunohistochemistry staining and immunofluorescence staining by confocal scanning. Images of the striatum around the ischemic area were captured. (a) TNF-*α*, (b) iNOS, (c) CD206, and (d) Arg-1. Sham: sham-operated rats, M + Veh: MCAO rats treated with vehicle, and M + GL: MCAO rats intragastrically administered with GL. Bar in the IHC image: 75 *μ*m and bar in the confocal image: 50 *μ*m.

Representative images of Iba-1, TNF-*α*, iNOS, CD206, and Arg-1 expression in the striatum surrounding the ischemic area are shown in Figure 4. The results showed that there was only weak Iba-1 immunoreactivity in the sham group, while the positive expression of Iba-1 was significantly enhanced in the vehicle group. The expression of Iba-1 was attenuated after GL treatment. However, since Iba-1 is a common marker of the activated microglia, it is difficult to distinguish which phenotype of microglia is activated. We, therefore, proceeded to perform the immunohistochemistry staining of M1 and M2 subtype markers of microglia, as well as their colocalization with Iba-1.

TNF-*α* and iNOS are markers of proinflammatory M1-type microglia. The results (Figures 4(a) and 4(b)) showed that the immunoreactivity of TNF-*α* and iNOS in the ischemic area of rats were significantly enhanced after CI/R, and most of the TNF-*α* and iNOS positive cells were colabeled with Iba-1. The results indicated that GL treatment could partially inhibit the activation of proinflammatory microglia.

CD206 and Arg-1 are markers of anti-inflammatoryM2-type microglia. The current study showed (Figures 4(c) and 4(d)) that CD206 and Arg-1 immunoreactivity increased in the ischemic periphery area, whereas activation of CD206 and Arg-1 labeled cells could be higher after GL treatment. The confocal immunofluorescence staining images showed not all Iba-1 positive cells were labeled with CD206 and Arg-1, but many CD206 and Arg-1 positive cells were labeled with Iba-1.

**Figure 5:**
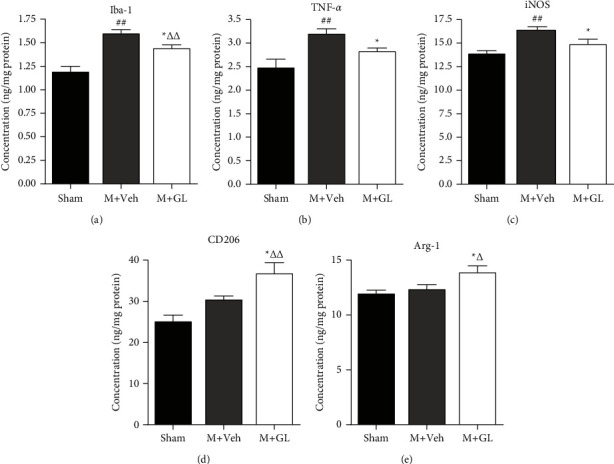
Effects of GL on the protein levels of microglia phenotype markers after cerebral ischemia/reperfusion injury. The ELISA method was used to detect the content of microglia phenotype markers in the brain tissue of the cerebral ischemia peripheral area. Data were obtained from 5 rats in the sham group and 7 rats in the other two groups. (a) Iba-1 concentration, (b) TNF-*α* concentration, (c) iNOS concentration, (d) CD206 concentration, and (e) Arg-1 concentration. Sham: sham-operated rats, M + Veh: MCAO rats treated with vehicle, and M + GL: MCAO rats intragastrically administered with GL. Results were presented as means ± SE. ^##^*P* < 0.01, M + Veh group compared with the sham group; ^*∗*^*P* < 0.05, M + GL group compared with the M + Veh group; ^Δ^*P* < 0.05 and ^ΔΔ^*P* < 0.01, M + GL group compared with the sham group.

To quantify the expression of these microglia phenotypic markers, ELISA was used to determine the protein levels of Iba-1, TNF-*α*, iNOS, CD206, and Arg-1. The results showed that the concentration of Iba-1, TNF-*α,* and iNOS increased significantly after CI/R injury (Figures 5(a)–5(c), *P* < 0.01), and GL treatment decreased the level of Iba-1 and TNF-*α* significantly (*P* < 0.05). CD206 and Arg-1 did not show statistically significant increases in ELISA (Figures 5(d) and 5(e)). However, GL treatment significantly enhanced the expression of CD206 and Arg-1 (*P* < 0.05).

**Figure 6:**
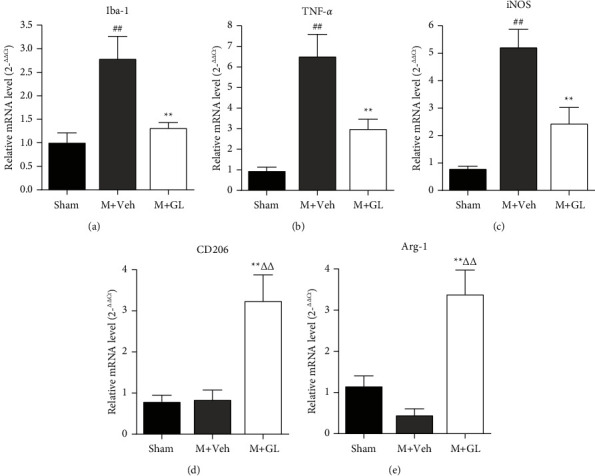
Effects of GL on the mRNA levels of microglia phenotype markers after cerebral ischemia/reperfusion injury. Data were obtained from 5 rats in the sham group and 7 rats in the other two groups. (a) Relative mRNA level of Iba-1, (b) relative mRNA level of TNF-*α*, (c) relative mRNA level of iNOS, (d) relative mRNA level of CD206, and (e) relative mRNA level of Arg-1. Sham: sham-operated rats, M + Veh: MCAO rats treated with vehicle, and M + GL: MCAO rats intragastrically administered with GL. Results were presented as means ± SE. ^##^*P* < 0.01, M + Veh group compared with the sham group; ^*∗*^*P* < 0.05 and ^*∗∗*^*P* < 0.01, M + GL group compared with the M + Veh group; ^ΔΔ^*P* < 0.01, M + GL group compared with the sham group.

We proceeded to evaluate the gene expression levels of these microglia phenotypes using RT-PCR. The results showed that the mRNA levels of Iba-1, TNF-*α,* and iNOS increased sharply after CI/R (Figures 6(a)–6(c), *P* < 0.01), and GL treatment could reduce the mRNA levels of those markers (Figures 6(a)–6(c), *P* < 0.01). For CD206 and Arg-1, cerebral ischemia injury did not elevate their gene expression levels. However, GL treatment can significantly enhance mRNA levels of CD206 and Arg-1 (Figures 6(d) and 6(e), *P* < 0.01).

## 4. Discussion

In the present study, we demonstrated that GL can significantly alleviate neurological deficits induced by focal CI/R. Using the ^18^F-FDG Micro-PET assay, we found that GL enhanced the glucose uptake in the brain, as well as Glut-1expression in the cerebral ischemic peripheral area. Therefore, the energy utilization of the brain was improved. Meanwhile, we observed that GL ameliorated the inflammatory response induced by CI/R and attenuated the abnormal activation of astrocytes and microglia. Further investigation of the activated microglia phenotype revealed that GL can regulate macrophage/microglia polarization, reducing markers of classical activation (M1) microglia, while increasing the markers of alternative pathway activation (M2) microglia. These results suggest that the effects of GL on neurobehavioral improvement in rats with CI/R injury may involve improved glucose utilization and inflammation.

It is well known that microglia, as macrophages in the CNS, constitute the main immune defense line in CNS [19]. After cerebral ischemia, the phenotype of macrophages/microglia changes from a resting state (M0 type) to an activated state (M1 or M2 type) [13]. Classical M1-type microglia play roles in proinflammation and neurotoxicity and inhibit axon growth. They also produce various proinflammatory factors, including IL-1*β*, IL-6, IL-12, IL-23, IFN-*γ,* and TNF-*α*. Inducible nitric oxide synthase (iNOS), COX-2, and some membrane surface molecules including CD16, CD32, and CD86 are major markers of M1-type microglia. The primary function of the alternatively activated M2-type microglia is to suppress the excessive inflammatory response, promote the survival of injured neurons, and reduce glia scar formation. The common surface markers of M2 microglia mainly include Arginase-1 (Arg-1), CD163, and CD206 [20]. Microglia exhibit distinct activation profiles according to the microenvironment and adaptively switch their phenotypes in a spatiotemporal manner in response to brain injury [21]. In the early stage of ischemia, M1-type microglia are activated, and their phenotypic markers begin to increase at 3 days after ischemia [22] and reach the maximum at 14 days. M1-type microglia dominate from 7 days after ischemia and last for several weeks. At this time, microglia promote the release of inflammatory factors, aggravate brain damage, and are not conducive to regeneration and repair in the later stage of cerebral ischemia. M2-type microglia increase rapidly within 24 hours after cerebral ischemia injury, and their phenotypic markers are highly expressed in the ischemic core area, generally reaching a peak 3–7 days after ischemia [23]. After that, M2 microglia gradually transformed into M1 microglia. At that time, M2-type microglia play a crucial role in alleviating neuronal injury, enhancing nerve regeneration and tissue repair, and attenuating neuronal apoptosis to a certain extent. In this study, considering that the efficacy of GL began to be remarkable 7 days after CI/R injury, we chose the 7-day time point to study the effect of GL on the phenotypes of microglia.

The benefits of modulating the microglia M1/M2 phenotype in neurological-related diseases have now attracted increasing attention [21]. Numerous studies demonstrate the importance of microglia transition from M1 to M2 in ischemic injury [24, 25]. There are several studies on the *in vitro* regulation of GL on microglia inflammatory injury [14, 26, 27]. On the lipopolysaccharide (LPS)- stimulated BV2 cell, the inhibitory effect of GL on the inflammatory response of microglia was confirmed. Our study demonstrated that GL reduced microglia activation in animal models of CI/R, while also detected the microglia polarization. The results showed that GL treatment after CI/R significantly decreased the mRNA and protein expressions of M1 microglia markers iNOS and TNF-*α*, while promoting the mRNA and protein expressions of M2 microglia markers CD206 and Arg-1, indicating that GL regulates the polarization direction of microglia. However, we found no statistically significant changes in the mRNA and protein expression of the M2-type microglia markers CD206 and Arg-1 seven days after CI/R. This may be due to the fading dynamic change of M2-type microglia at 7 days after ischemia. However, GL has a significant promoting effect on CD206 and Arg-1. This result suggests that GL can reduce proinflammatory factors and enhance anti-inflammatory factors after CI/R. Meanwhile, the anti-inflammatory effect of GL on CI/R injury is also reflected in the effect on abnormal activation of astrocytes. Astrocytes are predominant glial cells in the CNS, and glial fibrillary acidic protein (GFAP) is a hallmark intermediate filament protein in activated astrocytes [28]. Generally, the proliferation of reactive astrocytes is considered a specific manifestation of neuroinflammation. The current study found that the GL can significantly reverse the abnormal activation of astrocytes after cerebral ischemia (Figure 3(c)). Microglia and astrocytes are the main cell types that respond to various types of brain injury, and their functions are increasingly recognized as complex, often interacting (and sometimes even synergistic) [29]. Previous studies have shown that GL can reduce related neuroinflammatory factors after cerebral ischemia injury in rats. Our study suggests that GL may ameliorate inflammation after CI/R by altering the activation status of microglia and astrocyte.

Glucose is the main energy supply for the nervous system. Under normal circumstances, hexokinase is the main rate-limiting kinase of glucose metabolism, but in the case of cerebral ischemia and hypoxia, the main rate-limiting step of glucose utilization is glucose transport. Therefore, upregulation of Glut-1 and Glut-3 expression can alleviate energy exhaustion and exert neuroprotective effects. Glut may be a new target for the treatment of ischemic cerebrovascular disease [30]. In this study, ^18^F-FDG micro-PET/CT imaging detection showed that there was obvious low glucose uptake area in the brain after cerebral ischemia injury, indicating that ischemia reduces the viability of neural cells, resulting in decreased glucose uptake. GL can significantly reduce the volume of ^18^F-FDG low uptake area, suggesting that GL may enhance the uptake and utilization of glucose by neural cells after cerebral ischemia. Immunohistochemical staining showed that the expression of Glut-1 in brain tissue increased after cerebral ischemia, which may be an adaptive response of cerebral tissue after ischemia, in other words, increased glucose transportation to provide more energy. GL treatment significantly improved the expression of Glut-1, indicating that GL may enhance glucose uptake in brain tissue by elevating glucose transporter expression. This is the first report on the effect of GL on improving glucose uptake after cerebral ischemia. Recent research has shown [31] that improvements in the energy utilization status of brain tissue can modulate the polarization of microglia. M1 microglia are activated by canonical activation pathway LPS/IFN-*γ*, and their main metabolic pathways are anaerobic glycolysis and pentose phosphate, whilst M2 microglia are activated by IL-4/IL-13 and have an intact tricarboxylic acid (TCA) cycle and oxidative phosphorylation (OXPHOS) process with prominent fatty acid synthesis and metabolism. Our study found GL affects both glucose uptake and polarization of M1/M2 microglia after CI/R. This may be because GL alleviates cerebral ischemia injury by targeting multiple targets with multiple components. However, whether there is a correlation between these two effects and whether GL can specifically improve the energy metabolism of microglia and convert it into a phenotype that inhibits inflammation after cerebral ischemia is the focus of our further study.

## 5. Conclusion

Here, we report that GL significantly improves glucose metabolism, inhibits neuroinflammation-related microglia and astrocyte activation in the brain tissue after cerebral ischemia, and regulates the polarization of microglia by switching them into an anti-inflammatory type. We also illustrate that the effect of GL on ameliorating the neurological deficit after CI/R in rats may involve improvements in glucose energy utilization and inflammatory status. These results provided more experimental evidence for the clinical application of GL in the treatment of stroke sequelae.

## Figures and Tables

**Table 1:** 

Primer	Primer sequence (5′-3)	Length (bp)	Annealing temperature (°C)
Iba-1-S	ATGAGCCAGAGCAAGGATTT	163	60
Iba-1-A	AGCATTCGCTTCAAGGACA		60
R-TNF-*α*-S	CCAGGTTCTCTTCAAGGGACAA	80	60
R-TNF-*α*-A	GGTATGAAATGGCAAATCGGCT		60
R-INOS-S	TTCACGACACCCTTCACCACA	106	60
R-INOS-A	TCTCTGGGTCCTCTGGTCAAAC		60
R-Arg1-S	TCCAAGCCAAAGCCCATAGA	269	60
R-Arg1-A	GTTCCATTCTTCTGGGTCTCTGC		60
CD206-S	GGCAAATGTCCAGAGTCAGA	245	60
CD206-A	CTTTTAAGAGGTTCAACACGGT		60
R-GAPDH-S	CTGGAGAAACCTGCCAAGTATG	138	60
R-GAPDH-A	GGTGGAAGAATGGGAGTTGCT		60

## Data Availability

The data in the manuscript are from experiment results. More detailed supporting data can be provided upon request to the corresponding author.

## Abbreviations

Arg-1: Arginase-1
CCA: Common carotid artery
CI/R: Cerebral ischemia/reperfusion
CNS: Central nervous system
COX-2: Cyclooxygenase-2
ECA: External carotid artery
ELISA: Enzyme-linked immunosorbent assay
18F-FDG: 18F-fluorodeoxyglucose
FOV: Field of view
GFAP: Glial fibrillary acidic protein
GL: Gualou Guizhi decoction
Glut-1: Glucose transporter 1
Iba-1: Ionized calcium-binding adaptor molecule-1
ICA: Internal carotid artery
iNOS: Inducible nitric oxide synthase
IntDen: Integrated density
MCA: Middle cerebral artery
MCAO: Middle cerebral artery occlusion
mNSS: Modified neurological severity score
mRNA: Messenger ribonucleic acid
NMDA: N-methyl-D-aspartic acid
PET: Position emission tomography
RT-PCR: Reverse transcription-polymerase chain reaction
SUV: Standard uptake value
TNF-*α*: Tumor necrosis factor-*α*.

## References

[1] Paul S., Candelario-Jalil E. (2021). Emerging neuroprotective strategies for the treatment of ischemic stroke: an overview of clinical and preclinical studies. *Experimental Neurology*.

[2] Stinear C. (2010). Prediction of recovery of motor function after stroke. *The Lancet Neurology*.

[3] Hung J. W., Wu W. C., Chen Y. J., Pong Y. P., Chang K. C. (2021). Predictors of clinically important improvements in motor function and daily use of affected arm after a botulinum toxin A injection in patients with chronic stroke. *Toxins*.

[4] Zhang Y., Li H., Huang M. (2014). Neuroprotective effects of gualou guizhi decoction in vivo and in vitro. *Journal of Ethnopharmacology*.

[5] Chen X., Li H., Huang M. (2014). Effect of gua lou gui zhi decoction on focal cerebral ischemia-reperfusion injury through regulating the expression of excitatory amino acids and their receptors. *Molecular Medicine Reports*.

[6] Huang J., Tao J., Xue X. (2013). Gua lou gui zhi decoction exerts neuroprotective effects on post-stroke spasticity via the modulation of glutamate levels and AMPA receptor expression. *International Journal of Molecular Medicine*.

[7] Zhu X., Hu H., Li Z., Lin R., Mao J., Chen L. (2015). Gua lou gui zhi decoction attenuates post-stroke spasticity via the modulation of GABAB receptors. *Molecular Medicine Reports*.

[8] Hu H., Zhu X., Lin R., Li Z., Chen L. (2016). Suppressive effects of gua lou gui zhi decoction on MCAO-induced NO and PGE2 production are dependent on the MAPK and NF-*κ*B signaling pathways. *Molecular Medicine Reports*.

[9] Hu H., Li Z., Zhu X. (2013). Gua lou gui zhi decoction suppresses LPS-induced activation of the TLR4/NF-*κ*B pathway in BV-2 murine microglial cells. *International Journal of Molecular Medicine*.

[10] Mao J., Li Z., Lin R. (2015). Preconditioning with gua lou gui zhi decoction enhances H_2_O_2_-induced Nrf_2_/HO-1 activation in PC12 cells. *Experimental and Therapeutic Medicine*.

[11] Li M., Zhao Y., Zhan Y. (2020). Enhanced white matter reorganization and activated brain glucose metabolism by enriched environment following ischemic stroke: micro PET/CT and MRI study. *Neuropharmacology*.

[12] Lameka K., Farwell M. D., Ichise M. (2016). Positron emission tomography. *Handbook of Clinical Neurology*.

[13] Qin C., Zhou L. Q., Ma X. T. (2019). Dual functions of microglia in ischemic stroke. *Neuroscience Bulletin*.

[14] Chang X., Fang Y., Zhang Y. (2021). Gualou guizhi granule suppresses LPS-induced inflammatory response of microglia and protects against microglia-mediated neurotoxicity in HT-22 via akt/NF-*κ*B signaling pathways. *Evidence-based Complementary and Alternative Medicine*.

[15] Han J., Zhang J. Z., Zhong Z. F. (2018). Gualou guizhi decoction promotes neurological functional recovery and neurogenesis following focal cerebral ischemia/reperfusion. *Neural Regeneration Research*.

[16] Longa E. Z., Weinstein P. R., Carlson S., Cummins R. (1989). Reversible middle cerebral artery occlusion without craniectomy in rats. *Stroke*.

[17] Chen J., Li Y., Wang L. (2001). Therapeutic benefit of intravenous administration of bone marrow stromal cells after cerebral ischemia in rats. *Stroke*.

[18] Shohami E., Novikov M., Bass R. (1995). Long-term effect of HU-211, a novel non-competitive NMDA antagonist, on motor and memory functions after closed head injury in the rat. *Brain Research*.

[19] Xin W. Q., Wei W., Pan Y. L. (2021). Modulating poststroke inflammatory mechanisms: novel aspects of mesenchymal stem cells, extracellular vesicles and microglia. *World Journal of Stem Cells*.

[20] Xiong X. Y., Liu L., Yang Q. W. (2016). Functions and mechanisms of microglia/macrophages in neuroinflammation and neurogenesis after stroke. *Progress in Neurobiology*.

[21] Yu F., Huang T., Ran Y. (2021). New insights into the roles of microglial regulation in brain plasticity-dependent stroke recovery. *Frontiers in Cellular Neuroscience*.

[22] Zhu J., Cao D., Guo C. (2019). Berberine facilitates angiogenesis against ischemic stroke through modulating microglial polarization via AMPK signaling. *Cellular and Molecular Neurobiology*.

[23] Zhou S., Zhu W., Zhang Y., Pan S., Bao J (2018). S100B promotes microglia M1 polarization and migration to aggravate cerebral ischemia. *Inflammation Research*.

[24] Lu J., Wang J., Yu L. (2021). Treadmill exercise attenuates cerebral ischemia–reperfusion injury by promoting activation of M2 microglia via upregulation of interleukin-4. *Frontiers in Cardiovascular Medicine*.

[25] Tian R., Mao G. (2022). Ghrelin reduces cerebral ischemic injury in rats by reducing M1 microglia/macrophages. *European Journal of Histochemistry*.

[26] Hu H., Zhong X., Lin X., Yang J., Zhu X. (2021). Inhibitory effect of gualou guizhi decoction on microglial inflammation and neuron injury by promoting anti-inflammation via targeting mmu-miR-155. *Evidence-based Complementary and Alternative Medicine*.

[27] Hu H., Zhu X., Lin X. (2020). Gualou guizhi decoction represses LPS-induced BV2 activation via miR-155 induced inflammatory signals. *Pakistan journal of pharmaceutical sciences*.

[28] Hol E. M., Pekny M. (2015). Glial fibrillary acidic protein (GFAP) and the astrocyte intermediate filament system in diseases of the central nervous system. *Current Opinion in Cell Biology*.

[29] Magaki S. D., Williams C. K., Vinters H. V. (2018). Glial function (and dysfunction) in the normal & ischemic brain. *Neuropharmacology*.

[30] Espinoza-Rojo M., Iturralde-Rodriguez K. I., Chanez-Cardenas M. E., Ruiz-Tachiquin M. E., Aguilera P. (2010). Glucose transporters regulation on ischemic brain: possible role as therapeutic target. *Central Nervous System Agents in Medicinal Chemistry*.

[31] Yu S., Fu J., Wang J. (2021). The influence of mitochondrial-DNA-driven inflammation pathways on macrophage polarization: a new perspective for targeted immunometabolic therapy in cerebral ischemia-reperfusion injury. *International Journal of Molecular Sciences*.

